# Risk of Heart Failure in Patients with ST-Elevation Myocardial Infarction Receiving Drug-Eluting Stent Implantation and Undefined Duration of Antiplatelets

**DOI:** 10.3390/jpm12030369

**Published:** 2022-02-28

**Authors:** Li-Nien Chien, Chun-Chao Chen, Ya-Hui Chang, Fa-Chang Yu, Chen-Ting Tsai, Hung-Yi Liu, Hung-I Yeh, Chao-Feng Lin

**Affiliations:** 1 School of Health Care Administration, College of Management, Taipei Medical University, Taipei 110, Taiwan; lnchien@tmu.edu.tw (L.-N.C.); dieseabook@gmail.com (H.-Y.L.); 2 Health Data Analytics and Statistics Center, Office of Data Science, Taipei Medical University, Taipei 110, Taiwan; 3 Division of Cardiology, Department of Internal Medicine, Shuang Ho Hospital, Taipei Medical University, New Taipei City 235, Taiwan; b101092035@tmu.edu.tw; 4 Department of Medicine, MacKay Medical College, New Taipei City 252, Taiwan; yahui057@gamil.com (Y.-H.C.); hungi.yeh@msa.hinet.net (H.-I.Y.); 5 Department of Pharmacy, MacKay Memorial Hospital, Taipei 104, Taiwan; 6 Division of Cardiology, Department of Internal Medicine, MacKay Memorial Hospital, Taipei 104, Taiwan; buebueman0829@gmail.com (F.-C.Y.); chengtingtsai@gmail.com (C.-T.T.); 7 Ph.D. Program for Cancer Molecular Biology and Drug Discovery, College of Medical Science and Technology, Taipei Medical University and Academia Sinica, Taipei 115, Taiwan

**Keywords:** heart failure, ST-elevation myocardial infarction, drug-eluting stent, bare-metal stent

## Abstract

It remains unknown as to whether the use of new-generation drug-eluting stent (NG-DES) in patients with ST-elevation myocardial infarction (STEMI) who receive an undefined duration of dual antiplatelet therapy (DAPT) reduces the risk of hospitalization for heart failure (HHF). In this population-based retrospective cohort study, we applied propensity score matching to select 6831 pairs of patients with STEMI who had similar baseline characteristics and received either NG-DES or bare-metal stent (BMS) implantation between 1 January 2007 and 31 December 2016. The risk of stent-associated HHF was evaluated, wherein death was considered a competing risk. Rates of cumulative incidence competing risk for HHF at the 1, 2, 3, 4, and 5 year follow-up were lower in the NG-DES group (3.79%, 5.21%, 6.15%, 7.01%, and 8.29%, respectively) than in the BMS group (4.51%, 6.21%, 7.32%, 8.33%, and 9.83%, respectively). NG-DES implantation was associated with a lower risk of HHF than BMS implantation after 5 years, with an adjusted subdistribution hazard ratio of 0.82 (95% confidence interval 0.72–0.92, *p* = 0.001). These results accord with those of patients who received DAPT for >6 months. Our findings highlight that NG-DESs may reduce HHF risk in patients with STEMI receiving an undefined duration of DAPT.

## 1. Introduction

Heart failure (HF) after myocardial infarction (MI) is a strong predictor for all-cause death in patients with MI [1]. In a population-based cohort study [1], including a mean follow-up duration of 7.6 years, the mortality rates in patients who developed post-MI HF were 70%, whereas those in patients who did not develop post-MI HF were 28%. In addition, patients who had HF after discharge from the index MI admission had a worse outcome than did those patients who had HF during MI admission [1], indicating that a prevention strategy is required to reduce the long-term risk of HF after MI.

A 12 month duration of dual antiplatelet therapy (DAPT) is recommended in patients with ST-elevation MI (STEMI), while a short DAPT duration of 6 months is recommended in those patients who have features of high bleeding risk (HBR) [2,3]. In addition, the use of new-generation drug-eluting stent (NG-DES) in patients with STEMI has demonstrated superior benefits compared with that of bare-metal stent (BMS) in terms of target vessel failure (TVF) and repeat percutaneous coronary intervention (PCI) [2], all according to randomized controlled trial (RCT) [4,5,6] and meta-analysis [7,8] results. However, the aforementioned studies only investigated specific NG-DES types and did not measure the risk of new-onset HF after stent implantation as the principal study outcome [4,5,6,7,8]. In addition, the adherence of DAPT at 12 month follow-up in patients in the previous studies [4,5,6,7,8] was >90%, implying that the results could not be generalized to the patients suspected to have HBR along with uncertain DAPT compliance. Nonadherence to DAPT undoubtedly put patients at high risk of recurrent ischemic events, which potentially leads to the development of HF. Hence, in real clinical situations, it remains poorly understood as to whether the use of NG-DES, compared with that of BMS, is associated with a reduced long-term risk of HF after stent implantation in patients with STEMI who received undefined duration of DAPT.

The comparison between NG-DES and BMS in patients with STEMI is hardly addressed by RCTs because the use of NG-DES has become the recommended treatment strategy [2]. In Taiwan, the cost of NG-DES is paid by patients, whereas the cost of BMS is fully covered by the National Health Insurance (NHI). Although the cost of stents may influence the choice of the type of stent used, it provided an opportunity to compare the risk of stent-associated HF between NG-DES and BMS in patients with STEMI. Therefore, the current study investigated the effects of NG-DES implantation on the risk of hospitalization for HF (HHF) in patients with STEMI who received undefined duration of DAPT at the 5 year follow-up by using a real-world data from Taiwan’s NHI Research Database (NHIRD).

## 2. Materials and Methods

### 2.1. Data Source

This study was approved by the Institutional Review Board of MacKay Memorial Hospital (MMH-IRB; approval no. 20MMHIS351e). NHIRD is a database that contains the claims data of 99% of Taiwan’s residents covered by NHI. Because individual identifiers in the NHIRD have been encrypted and cannot be recognized, the need for informed consent was waived under the full review process of the MMH-IRB. The NHIRD includes data on inpatient, outpatient, and prescription drug claims. The prescribed medications can be classified using the Anatomical Therapeutic Chemical (ATC) system; disease diagnoses are coded according to the International Classification of Diseases, Ninth Revision, Clinical Modification (ICD-9-CM) until 2016 and according to the International Classification of Diseases, Tenth Revision (ICD-10), subsequently. Death records from the National Death Registry are linked to the NHIRD on the basis of patients’ encrypted identifiers [9].

### 2.2. Study Cohort

**Figure 1:**
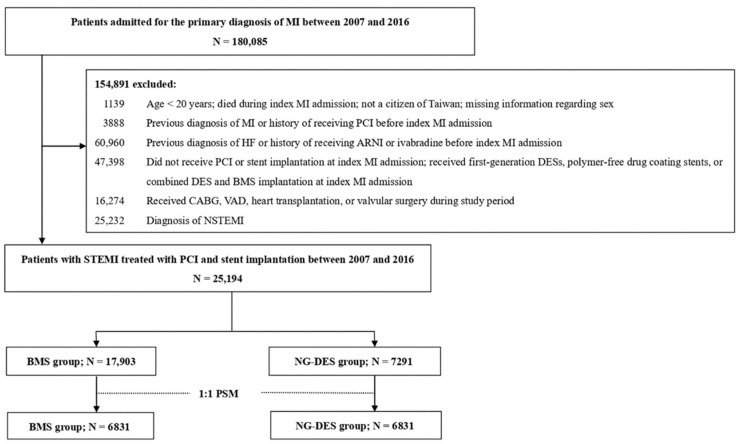
Patient selection process. ARNI—angiotensin receptor/neprilysin inhibitor; BMS—bare-metal stent; CABG—coronary artery bypass grafting; DES—drug-eluting stent; HF—heart failure; MI = myocardial infarction; N—number; NG-DES—new-generation drug-eluting stent; NSTEMI—non-ST-elevation myocardial infarction; PCI—percutaneous coronary intervention; PSM—propensity score matching; STEMI—ST-elevation myocardial infarction; VAD—ventricular assist device.

In the retrospective cohort, we included patients who were admitted for a primary diagnosis of MI (ICD-9-CM 410 and ICD-10 I21.0-I21.4, I22.0-I22.2, I22.8, and I22.9) between 2007 and 2016. The date of the first MI admission was considered the index date of MI. We excluded patients who were aged <20 years, were not Taiwanese citizens, had died during admission, had a previous MI or HF diagnosis, had received ivabradine or angiotensin receptor/neprilysin inhibitor (the recommended medications for chronic HF with systolic dysfunction) [10], had received PCI before admission, did not receive PCI or stent implantation, or had received polymer-free drug-coating stent or combined DES and BMS implantation at index MI admission. We also excluded patients who received first-generation DES implantation, which is associated with an increased stent thrombosis risk [11], and in turn, raises safety concerns and leads to a considerably decreased use in Taiwan. Moreover, patients with high HF risk, including those who received coronary artery bypass grafting [12], ventricular assist device support [13], valvular surgery [14], or heart transplantation [15] during the study period were excluded. Finally, patients who received a non-STEMI diagnosis were excluded. Figure 1 illustrates the patient selection process.

### 2.3. Propensity Score Matching

To reduce potential bias in patient selection, we used propensity score matching (PSM) to select pairs of patients with similar baseline characteristics but different implanted stent types (i.e., NG-DES or BMS). PSM is being increasingly used in studies estimating the treatment effects by employing observational data [16]. In the present study, pairs of patients were matched on the basis of the logit of the propensity score by using calipers of widths equal to 0.2 of the standard deviation.

Covariates used to calculate the propensity score are listed in Table 1: age, sex, index MI admission year, comorbidities, prescribed medications, multivessel PCI use, intraaortic balloon pump (IABP) use, CHA_2_DS_2_-VASc score (including congestive HF, hypertension, age ≥75 years, diabetes, stroke/transient ischemic attack, vascular disease, age = 65–74 years, female sex) [17], ORBIT score (including age ≥75 years, insufficient kidney function, treatment with any antiplatelet, a positive clinical history of bleeding, and anemia or abnormal hemoglobin) [18], and Academic Research Consortium (ARC)-defined major (including history of malignancy, use of anticoagulants, end-stage chronic kidney disease, and intracranial hemorrhage) and minor (including age ≥75 years, history of ischemic stroke, and history of bleeding events requiring hospitalization or transfusion) bleeding risk criteria [19].

Comorbidities were defined if patients had two or more diagnostic claims within 1 year prior to the index MI admission. Prescribed medications included angiotensin-converting enzyme inhibitors (ACEIs) or angiotensin II receptor blockers (ARBs), beta-blockers, nitrates, aspirin, P2Y_12_ inhibitors, statins, proton pump inhibitors (PPIs), steroids, and nonsteroidal anti-inflammatory drugs (NSAIDs). The diagnostic codes for diseases and the ATC codes for medications are listed in Table S1.

We used multivessel PCI and CHA_2_DS_2_-VASc scores as indicators to represent the severity of coronary artery disease (CAD) [20]. To consider the potential risk of bleeding complications, we referred to patients who had at least one major or two minor ARC criteria for bleeding risk as patients with ARC-HBR according to the ARC’s definition [19]. We also used the ORBIT score to assist the assessment of patients’ bleeding risk, in which patients with ORBIT scores of 0–2 were classified as low risk, whereas those with ORBIT scores of ≥3 were classified as medium to high risk [18].

### 2.4. Study Outcomes

The main study outcome was the occurrence of HHF (ICD-9-CM code 428 and ICD-10 code I50) after stent implantation. The study patients were followed up for 5 years. Those who died before developing HF during the follow-up period were considered to have competing risks.

### 2.5. Statistical Methods

Primary analysis was performed to examine the risk of HHF associated with the use of the two stent types (i.e., NG-DES and BMS) in patients with STEMI. The consistency of the treatment effect was evaluated among eight prespecified subgroups stratified by age, sex, number of treated vessels, number of implanted stents, use of an IABP, CHA_2_DS_2_-VASc scores, ORBIT scores, and whether the patients were at ARC-HBR [19]. We used a competing risk model in which death was considered a competing risk and reported the adjusted subdistribution hazard ratio (SHR) to account for the possibility that patients might have died before HHF occurrence. The variables listed in Table 1 were used in the competing risk model when examining the association between implanted stent types and the occurrence of HHF. To further examine the abovementioned association in patients with STEMI who were compliant with guideline-recommended duration of DAPT [2,3], we also performed a separate analysis of patients who received DAPT for an undefined duration of >6 months after the index MI admission. All analyses were performed using SAS/STAT 9.4 (SAS Institute, Cary, NC, USA) and STATA 14 software (Stata Corp, College Station, TX, USA). A standardized mean difference (SMD) >0.1 indicated imbalance between the two groups, and a *p* of <0.05 indicated a significant association between risk of HHF and type of stent used.

## 3. Results

Of the 25,194 patients with STEMI who received PCI and stent implantation included in this study, 7291 (28.9%) received NG-DES implantation. The patients who were aged ≥75 years had less frequently received NG-DES implantation than those patients who were <75 years. Overall, the use of NG-DESs gradually increased over time. Compared with patients in the BMS group, those in the NG-DES group had a higher prevalence of dyslipidemia; had a lower prevalence of cerebrovascular disease; had more frequently used beta-blockers and statins; had less frequently used PPIs, steroids, and NSAIDs; had less frequently used IABP; and had lower ORBIT scores (Table 1). Using PSM, we selected 6831 patient pairs, and the baseline characteristics in the NG-DES and BMS groups were highly similar (Table 1).

**Figure 2:**
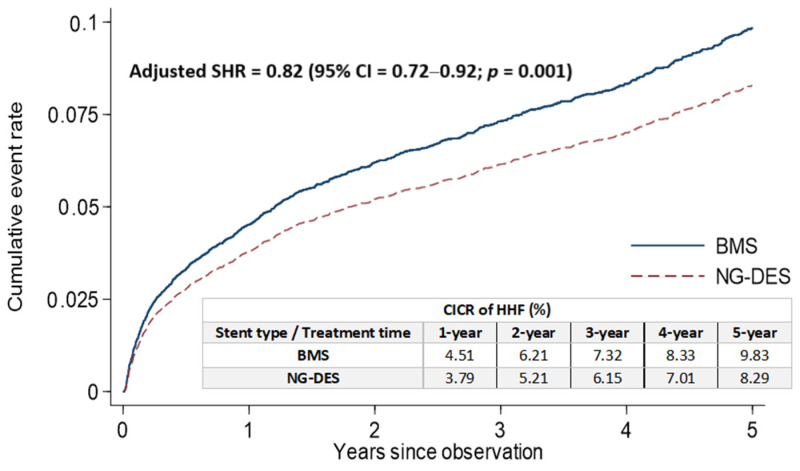
Cumulative incidence of HHF for competing risk among patients with STEMI receiving NG-DES or BMS implantation. BMS—bare-metal stent; CI—confidence interval; CICR—cumulative incidence competing risk; HHF—hospitalization for heart failure; NG-DES—new-generation drug-eluting stent; SHR—subdistribution hazard ratio; STEMI—ST-elevation myocardial infarction.

The rates of cumulative incidence competing risk (CICR) of HHF after stent implantation at the 1, 2, 3, 4, and 5 year follow-up were lower in the NG-DES group (3.79%, 5.21%, 6.15%, 7.01%, and 8.29%, respectively) than in the BMS group (4.51%, 6.21%, 7.32%, 8.33%, and 9.83%, respectively; Figure 2). After adjustments for covariates listed in Table 1, NG-DES implantation in patients with STEMI was associated with a decreased HHF risk at the 5 year follow-up compared with BMS implantation (adjusted SHR 0.82, 95% confidence interval (CI) 0.72–0.92, *p* = 0.001; Figure 2). We also conducted an analysis based on types of NG-DESs categorized by their coated drugs (Table S2) and observed that the rates of CICR and the risk of stent-associated HHF were comparable among different NG-DESs.

**Figure 3:**
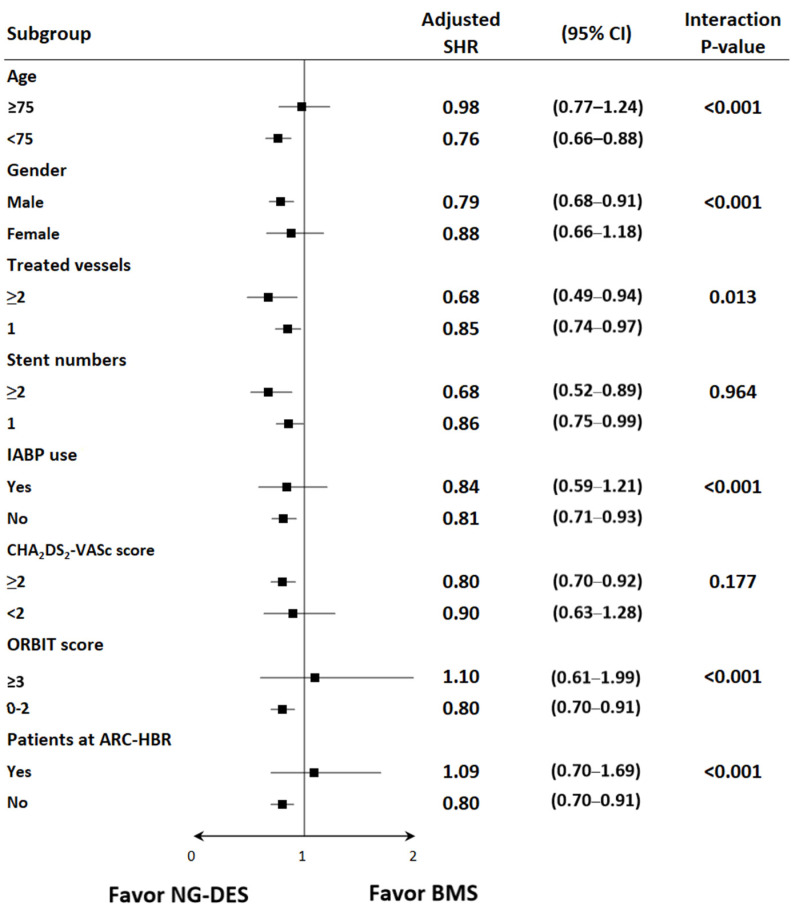
Subgroup analysis of the risk of HHF among patients with STEMI receiving NG-DESs (reference group) and BMSs. Adjusted SHR was adjusted for baseline covariates, including age, sex, year of index MI admission, comorbidities, prescribed medications, use of IABP during PCI, multivessel PCI, CHA_2_DS_2_-VASc scores, ORBIT scores, and various ARC criteria for HBR. Abbreviations are defined in the footnote of Table 1.

The subgroup analysis showed that the benefits of NG-DESs over BMSs were consistent in the following prespecified groups: patients who received two or more stents versus those who received one stent and patients with a CHA_2_DS_2_-VASc score of ≥2 versus those with a CHA_2_DS_2_-VASc score of <2 (Figure 3). Meanwhile, patients with STEMI who were aged <75 years, male, and received PCI for two or more vessels did not use IABP, had ORBIT scores <3, and were not at ARC-HBR received more benefits from NG-DESs than did their counterparts (Figure 3).

After excluding patients who had received <6 months of DAPT, received no DAPT, died, or had a diagnosis of HF within 6 months after the index MI, we generated a second cohort comprising those who had received DAPT for >6 months after stent implantation (Figure S1). Baseline characteristics of the NG-DES and BMS groups in the second cohort were similar after using PSM (Table S3). Of the patients who received DAPT for >6 months after stent implantation, the CICR of HHF at 1, 2, 3, 4, and 5 year follow-ups remained lower in the NG-DES group (1.81%, 2.96%, 3.86%, 4.94%, and 6.02%, respectively) than in the BMS group (2.34%, 3.82%, 4.99%, 6.38%, and 7.75%, respectively; adjusted SHR 0.78, 95% CI 0.62–0.97, *p* = 0.024; Figure S2). In addition, the results of subgroup analysis in the second cohort were similar to those in the original cohort (Figure S3).

## 4. Discussion

In our knowledge, the present study is the first real-world evidence using a large-scale, population-based database to demonstrate that NG-DES use, compared with BMS use, is associated with a reduced risk of HHF among patients with STEMI at the 5 year follow-up. The Harmonizing Outcomes with Revascularization and Stents in Acute Myocardial Infarction (HORIZONS-AMI) trial has reported that HF incidence rate in patients with STEMI was 5.2% at the 2 year follow-up [21]. However, the HORIZONS-AMI trial reported a relative short-term observation in patients who received specific types of first-generation DES and BMS [22], thereby hardly inferring the long-term effect of NG-DES implantation on the risk of HHF in patients with STEMI. Our present study demonstrated not only a long-term benefit of NG-DES in reducing the risk of HHF but also a decreased risk of repeat PCI (Table S4) and recurrent MI (Table S5) associated with NG-DES implantation, which may limit adverse myocardial remodeling and explain our findings.

Although a 6 month duration of DAPT is considered in patients with STEMI who exhibit HBR features, irrespective of the implanted stent type [2,3,23], the choice of stent types for patients with STEMI deemed to have HBR is challenging because of the lack of consensus-based definition of HBR and the patients’ uncertain compliance with DAPT [24]. A recent meta-analysis [24] analyzed three landmark RCTs [25,26,27] and determined that NG-DES with 1 month DAPT in patients with various high-risk features of bleeding complications lowered the risk of adverse events compared with BMS implantation. However, only a small portion of patients (4–15%) was identified as having STEMI in these aforementioned RCTs [25,26,27]. In addition, the criteria to define HBR used in each trial were quite different [25,26,27], which resulted in poor generalizability to real-world scenarios. In this study, we did not observe the superiority of NG-DES over BMS when the analyses focused on patients with STEMI who were at ARC-HBR identified by the ARC’s definition. Our results were similar to those of patients with STEMI deemed to have various high-risk features of bleeding complications in the previous study [28]. Patients who were at ARC-HBR in our study cohort were older in age and had more comorbidities compared with those who were not at ARC-HBR (Table S6), which might lead to a higher risk of developing HF and counteract the benefits of NG-DES. Further RCTs using a consensus-based definition of HBR are needed to determine which type of stent is favored in patients with STEMI who are at HBR.

Approximately 50% of patients with STEMI have multivessel disease in which the current guidelines have recommended that PCI for non–infarct-related arteries can be considered in selected patients [2,29]. Recently, the Complete Revascularization with Multivessel PCI for Myocardial Infarction (COMPLETE) trial [30] showed that complete revascularization with multivessel PCI was superior to IRA-only PCI in reducing adverse cardiovascular outcomes among patients with STEMI and multivessel disease. However, the COMPLETE trial failed to demonstrate which type of stents was favored in multivessel PCI strategy. The data presented here could provide further implication for considering NG-DESs to improve clinical outcomes in patients with STEMI who received multivessel PCI strategy and multiple stent implantation.

In the subgroup analysis, we did not observe the superiority of NG-DES over BMS in older and female patients. Female patients with STEMI tend to have an increased extent of endothelial inflammation and microvascular dysfunction and have more baseline risk profiles, such as diabetes, hypertension, obesity, and psychological distress, which may influence the risk of HF to a larger degree compared with male patients with STEMI [31]. In addition, elderly patients may frequently have contraindications or intolerance to ACEIs, ARBs, and beta-blockers that are guideline-directed medications for HF [10]. These aforementioned differences may contribute to sex and age disparities in the benefits of NG-DES.

The strength of this study is the use of large-scale real-world data to investigate a prominent concern that is hardly addressed using RCTs or meta-analyses in the contemporary era. We also used PSM to reduce the potential patient selection bias. Moreover, in our analyses, we considered various high-risk characteristics of bleeding complications, including the ORBIT scores and ARC criteria for HBR, which were rarely included in previous studies, so that we could observe the influence of HBR features on stent-associated HF risk in patients with STEMI who received undefined duration of DAPT.

This study has some limitations. First, even when PSM is applied, a nonrandomized retrospective design may introduce bias because of some confounders, such as patients’ baseline left ventricular ejection fraction and serum brain natriuretic peptide levels, angiographic findings of PCI, and the extent of patients’ CAD that could not be measured in the claim-based data. Moreover, the NHIRD did not provide certain clinical information, such as stent thrombosis and TVF, that might influence the occurrences of HHF. In the present study, we showed that the use of NG-DES was superior to BMS in terms of a reduced risk of repeat PCI (Table S4) and recurrent MI (Table S5), which might be two proxy indicators of stent thrombosis and TVF. A long period of observation in our present study, which included heterogeneity of patient composition and treatment methods, might also have introduced some bias in our results. Second, the implementation of PSM undoubtedly reduced the external validity of our study because only a subset of treated patients was analyzed; however, despite its limitations, PSM remains an acceptable approach that has been validated in RCTs [32]. Finally, the study cohort was limited to an Asian population; therefore, the results might not be applicable to other populations. Future prospective randomized studies are warranted to confirm our findings.

## 5. Conclusions

The NG-DES implantation exhibited a superiority over BMS in reducing the 5 year risk of HHF among patients with STEMI who received undefined duration of DAPT; the results are consistent with those for patients who had received DAPT for >6 months. Overall, these real-world data suggest that NG-DESs significantly prevent HHF after PCI in patients with STEMI.

## Figures and Tables

**Table 1:** Baseline characteristics of patients with STEMI receiving NG-DES or BMS before and after PSM.

	Before PSM					After PSM				
	NG-DES		BMS		SMD *	NG-DES		BMS		SMD *
	*n*	(%)	*n*	(%)		*n*	(%)	*n*	(%)	
N (%)	7291		17,903			6831		6831		
Age, mean (SD)	59.0 (12.5)		60.5 (13.4)			59.2 (12.4)		59.2 (12.4)		
Age group, *n* (%)										
20–44	873	(12.0)	1993	(11.1)	0.026	775	(11.3)	780	(11.4)	0.002
45–64	4144	(56.8)	9354	(52.2)	0.092	3881	(56.8)	3881	(56.8)	<0.001
65–74	1341	(18.4)	3399	(19.0)	0.015	1287	(18.8)	1282	(18.8)	0.002
≥75	933	(12.8)	3157	(17.6)	0.135	888	(13.0)	888	(13.0)	<0.001
Male (%)	8935	(81.4)	15,086	(84.3)	0.069	5989	(87.7)	5989	(87.7)	<0.001
Clinical data of index PCI										
Diagnostic year										
2007–2010	873	(12.0)	7504	(41.9)	0.717	868	(12.7)	868	(12.7)	<0.001
2011–2013	2334	(32.0)	5567	(31.1)	0.020	2279	(33.4)	2279	(33.4)	<0.001
2014–2016	4084	(56.0)	4832	(27.0)	0.616	3684	(53.9)	3684	(53.9)	<0.001
Multivessel PCI	846	(11.6)	1661	(9.3)	0.076	715	(10.5)	586	(8.6)	0.064
Number of stents, mean (SD)	1.23 (0.53)		1.28 (0.57)			1.23 (0.53)		1.23 (0.52)		
IABP use, yes, *n* (%)	368	(5.3)	1761	(9.8)	0.172	375	(5.5)	364	(5.3)	0.007
Comorbidity										
DM	2122	(29.1)	5537	(30.9)	0.040	2006	(29.4)	1988	(29.1)	0.006
HTN	3921	(53.8)	9326	(52.1)	0.034	3655	(53.5)	3636	(53.2)	0.006
Dyslipidemia	3784	(51.9)	7927	(44.3)	0.153	3523	(51.6)	3440	(50.4)	0.024
CVD	373	(5.1)	1391	(7.8)	0.108	362	(5.3)	351	(5.1)	0.007
AF	217	(3.0)	593	(3.3)	0.019	201	(2.9)	206	(3.0)	0.004
COPD/asthma	306	(4.2)	962	(5.4)	0.055	298	(4.4)	284	(4.2)	0.010
Dementia/parkinsonism	76	(1.0)	359	(2.0)	0.079	72	(1.1)	72	(1.1)	<0.001
OA/RA/rheumatism	697	(9.6)	1906	(10.6)	0.036	656	(9.6)	656	(9.6)	<0.001
CHA_2_DS_2_-VASc score										
≥2	5478	(75.1)	13,771	(76.9)	0.042	5121	(75.0)	5115	(74.9)	0.002
0–1	1813	(24.9)	4132	(23.1)	0.042	1710	(25.0)	1716	(25.1)	0.002
Medication use										
ACEI/ARB	5876	(80.6)	14,128	(78.9)	0.042	5510	(80.7)	5476	(80.2)	0.013
Beta-blockers	5661	(77.6)	12,623	(70.5)	0.163	5240	(76.7)	5203	(76.2)	0.013
Nitrates	67,32	(92.3)	16,168	(90.3)	0.072	6297	(92.2)	6174	(90.4)	0.064
Aspirin	7243	(99.3)	17,695	(98.8)	0.053	6784	(99.3)	6792	(99.4)	0.015
P2Y12 inhibitors	7271	(99.7)	17,814	(99.5)	0.036	6811	(99.7)	6818	(99.8)	0.021
Statins	6271	(86.0)	13,028	(72.8)	0.332	5840	(85.5)	5861	(85.8)	0.009
PPIs	755	(10.4)	2501	(14.0)	0.111	709	(10.4)	750	(11.0)	0.019
Steroids	687	(9.4)	2414	(13.5)	0.128	667	(9.8)	701	(10.3)	0.017
NSAIDs	2000	(27.4)	6543	(36.5)	0.196	1940	(28.4)	1784	(26.1)	0.051
ORBIT score										
≥3	179	(2.5)	821	(4.6)	0.116	173	(2.5)	182	(2.7)	0.008
0–2	7112	(97.5)	17,082	(95.4)	0.116	6658	(97.5)	6649	(97.3)	0.008
ARC criteria of bleeding risk										
Major criteria, yes										
Malignancy	183	(2.5)	513	(2.9)	0.022	172	(2.5)	181	(2.6)	0.008
Long-term use of anticoagulants	98	(1.3)	354	(2.0)	0.050	95	(1.4)	100	(1.5)	0.006
End-stage CKD	15	(0.2)	55	(0.3)	0.020	15	(0.2)	19	(0.3)	0.012
ICH	36	(0.5)	150	(0.8)	0.042	34	(0.5)	38	(0.6)	0.008
Minor criteria, yes										
Age ≥ 75	933	(12.8)	3157	(17.6)	0.135	888	(13.0)	888	(13.0)	<0.001
Ischemic stroke	207	(2.8)	796	(4.4)	0.086	198	(2.9)	201	(2.9)	0.003
Bleeding events requiring hospitalization or transfusion	90	(1.2)	444	(2.5)	0.092	88	(1.3)	103	(1.5)	0.019
DAPT at discharge of index MI										
Aspirin	7228	(99.1)	17,654	(98.6)	0.050	6770	(99.1)	6779	(99.2)	0.015
P2Y12 inhibitors	7271	(99.7)	17,811	(99.5)	0.038	6811	(99.7)	6818	(99.8)	0.021

ACEI—angiotensin-converting enzyme inhibitor; ARB—angiotensin II receptor blocker; ARC—Academic Research Consortium; AF—atrial fibrillation; BMS—bare-metal stent; CHA_2_DS_2_-VASc score—congestive heart failure, hypertension, age ≥ 75 years, diabetes, stroke/transient ischemic attack, vascular disease, age 65 to 74 years, female sex; CKD—chronic kidney disease; COPD—chronic obstructive pulmonary disease; CVD—cerebrovascular disease; DES—drug-eluting stent; DM—diabetes mellitus; HHF—hospitalization for heart failure; HTN—hypertension; IABP—intraaortic balloon pump; ICH—intracranial hemorrhage; NG-DES—newer-generation drug-eluting stent; NSAIDs—nonsteroidal anti-inflammatory drugs; OA—osteoarthritis; OACs—oral anticoagulants; ORBIT score—age ≥ 75 years, bleeding history, chronic kidney disease, treatment with antiplatelet; PCI—percutaneous coronary intervention; PPIs—proton pump inhibitors; PSM = propensity score matching; RA—rheumatoid arthritis; SD—standard deviation; SMD—standardized mean difference; STEMI—ST-elevation myocardial infarction. *—difference in means or proportions divided by standard error and imbalance defined as an absolute value >0.1.

## Data Availability

The datasets used and/or analyzed during the current study are available from the corresponding author on reasonable request.

## Supplementary Materials

The following supporting information can be downloaded at https://www.mdpi.com/article/10.3390/jpm12030369/s1. Table S1: Disease diagnostic coding and ATC code for medication. Table S2: The rates of CICR and the risk of stent-associated HHF between different NG-DESs categorized by coated drugs. Figure S1: Selection process of patients with STEMI who received DAPT for an undefined duration of longer than 6 months following stent implantation. Table S3: Baseline characteristics of patients with STEMI who received DAPT for an undefined duration of longer than 6 months following stent implantation. Figure S2: Cumulative incidence of HHF for competing risk among patients with STEMI who received DAPT for an undefined duration of longer than 6 months following stent implantation. Figure S3: Subgroup analysis of the risk of HHF among patients with STEMI who received DAPT for an undefined duration of longer than 6 months following stent implantation. Table S4: The rates of CICR and the risk of repeat PCI in patients with STEMI receiving NG-DESs compared with those patients with STEMI receiving BMSs. Table S5: The rates of CICR and the risk of recurrent MI in patients with STEMI receiving NG-DESs compared with those patients with STEMI receiving BMSs. Table S6: Baseline characteristics of patients with STEMI who were at ARC-HBR and non-ARC-HBR after PSM.
