# Supplementary material for: Risk of Heart Failure in Patients with ST-Elevation Myocardial Infarction Receiving Drug-Eluting Stent Implantation and Undefined Duration of Antiplatelets
Source: J Pers Med. 2022 Feb 28;12(3):369. doi: 10.3390/jpm12030369 (PMC8950168; doi:10.3390/jpm12030369)
Supplement: Supplementary file 1 [file jpm-12-00369-s001.zip › jpm-1574116-supplementary.pdf]

Supplementary Materials

**Table S1. Disease diagnostic coding and ATC code for medication**	
**Disease/Comorbidity**	**ICD-9-CM/ATC**
MI	410
STEMI	410.0, 410.1, 410.2, 410.3, 410.4, 410.5, 410.6, 410.8
NSTEMI	410.7, 410.9
HF	428
DM	250
HTN	401–402
Dyslipidemia	272
CVD	430–438
AF	427.31
Cancer	140–239
COPD	491, 492, 494, 496
Asthma	493
Dementia	290–294
Parkinsonism	332
OA	715
RA	714
Rheumatism	729
Bleeding	535.01, 535.11, 535.21, 535.31, 535.41, 535.51, 535.61, 537.83, 562.02, 562.03, 562.12, 562.13, 569.3, 569.85, 578.0, 578.1, 578.9, 430, 431, 432, 432.0, 432.1, 432.9, 767.0, 852–852.5, 853–853.1
CRD	582, 583–583.7, 585, 586, 587, 588
**Medications**	
ACEI/ARB	C09AA, C09BA, C09BB, C09CA, C09DA, C09DB, C09DX
ARNI	C09DX04
Beta-blocker	C07AA, C07AB, C07AG
Ivabradine	C01EB17
Nitrate	C01DA, C01DX
Aspirin	B01AC06
$P2Y_{12}$ inhibitors	B01AC04 (Clopidogrel), B01AC22 (Prasugrel), B01AC24 (Ticagrelor)
Statin	C10AA
PPI	A02BC
Steroid	H02, R03BA
NSAID	M01A, M02A, N02BA
OAC	Warfarin (B01AA03), Rivaroxaban (B01AF01), Apixaban (B01AF02), Dabigatran (B01AE07), Edoxaban (B01AF03)

Abbreviations: ACEI = angiotensin-converting enzyme inhibitor; AF = atrial fibrillation; ARB = angiotensin II receptor blocker; ARNI = angiotensin receptor/neprilysin inhibitor; ATC = anatomical therapeutic chemical; COPD = chronic obstructive pulmonary disease; CRD = chronic renal disease; CVD = cerebrovascular disease; DM = diabetes mellitus; HTN = hypertension; ICD-9-CM = International Classification of Diseases, Ninth Revision, Clinical Modification; MI = myocardial infarction; NSAID = nonsteroidal anti-inflammatory drug; NSTEMI = non–ST-elevation myocardial infarction; OA = osteoarthritis; OAC = oral anticoagulant; PPI = proton pump inhibitor; RA = rheumatoid arthritis; STEMI = ST-elevation myocardial infarction.

Table S2. The rates of CICR and the risk of stent-associated HHF between different NG-DESs categorized by coated drugs.

NG-DESs / Follow-ups	1-year	2-year	3-year	4-year	5-year	Adjusted SHR (95% CI)	*p*
**SES**	5.09%	6.98%	8.20%	9.42%	11.3%	Reference	-
**EES**	3.41%	4.70%	5.53%	6.36%	7.66%	0.66 (0.43 – 1.02)	0.06
**BES**	3.65%	5.02%	5.91%	6.80%	8.18%	0.71 (0.46 – 1.11)	0.13
**ZES**	3.93%	5.41%	6.36%	7.31%	8.80%	0.77 (0.51 – 1.16)	0.21

Abbreviation: BES = biolimus-eluting stent; CI = confidence interval; CICR = cumulative incidence competing risk; EES = everolimus-eluting stent; HHF = hospitalization for heart failure; NG-DES = new-generation drug-eluting stent; SES = sirolimus-eluting stent; SHR = subdistribution hazard ratio; ZES = zotarolimus-eluting stent.

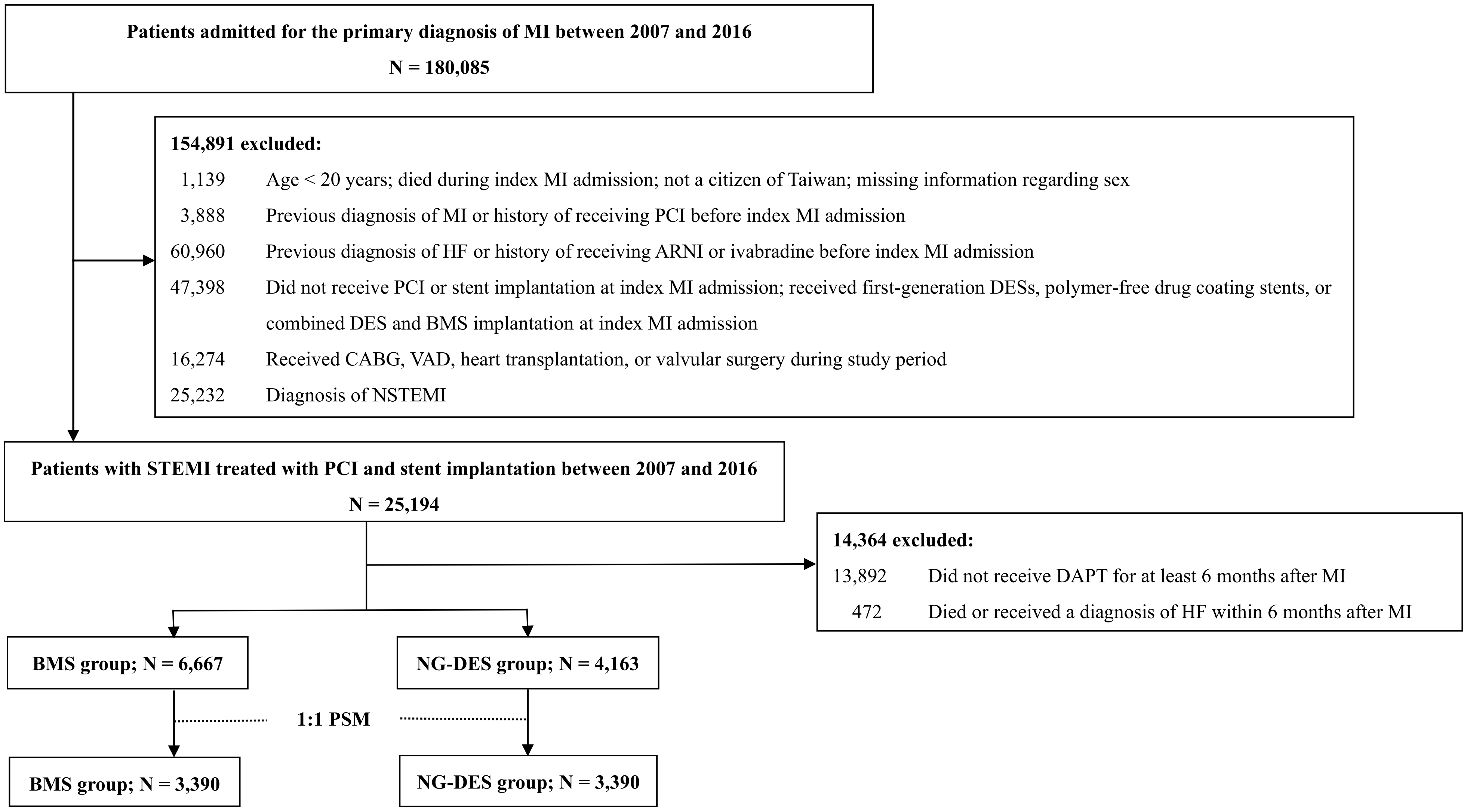

Figure S1. Selection process of patients with STEMI who received DAPT for an undefined duration of longer than 6 months following stent implantation. Abbreviations: ARNI = angiotensin receptor/neprilysin inhibitor; BMS = bare-metal stent; CABG = coronary artery bypass graft; DAPT = dual antiplatelet therapy; DES = drug-eluting stent; HF = heart failure; MI = myocardial infarction; NG-DES = new-generation drug-eluting stent; NSTEMI = non–ST-elevation myocardial infarction; PCI = percutaneous coronary intervention; PSM = propensity score matching; STEMI = ST-elevation myocardial infarction; VAD = ventricular assist device.

Table S3. Baseline characteristics of patients with STEMI who received DAPT for an undefined duration of longer than 6 months following stent implantation.										
	**Before PSM**					**After PSM**				
	**NG-DES**		**BMS**		**SMD***	**NG-DES**		**BMS**		**SMD***
	n	(%)	n	(%)		n	(%)	n	(%)	
N (%)	4,163		6,667			3,390		3,390		
**Age, mean (SD)**	58.1 (11.8)		58.6 (12.6)			58.1 (11.5)		58.1 (11.5)		
**Age group, n (%)**										
20–44	518	(12.4)	845	(12.7)	0.007	404	(11.9)	407	(12.0)	0.003
45-64	2,491	(59.8)	3,807	(57.1)	0.056	2,051	(60.5)	2,037	(60.1)	0.008
65–74	739	(17.8)	1,149	(17.2)	0.014	618	(18.2)	634	(18.7)	0.012
≥75	415	(10.0)	866	(13.0)	0.095	317	(9.4)	312	(9.2)	0.005
**Male (%)**	3,651	(87.7)	5,766	(86.5)	0.036	3,039	(89.6)	3,039	(89.6)	<0.001
**Clinical data of index PCI**										
Diagnostic year										
2007–2010	446	(10.7)	2,291	(34.4)	0.590	428	(12.6)	428	(12.6)	<0.001
2011–2013	1,345	(32.3)	2,294	(34.4)	0.045	1,236	(36.5)	1,236	(36.5)	<0.001
2014–2016	2,372	(57.0)	2,082	(31.2)	0.537	1,726	(50.9)	1,726	(50.9)	<0.001
Multivessel PCI	454	(10.9)	579	(8.7)	0.075	318	(9.4)	258	(7.6)	0.064
Number of stents, mean (SD)	1.23 (0.54)		1.29 (0.58)			1.24 (0.54)		1.25 (0.54)		
IABP use, yes, n (%)	190	(4.6)	561	(8.4)	0.157	169	(5.0)	163	(4.8)	0.008
**Comorbidity**										
DM	1,153	(27.7)	1,988	(29.8)	0.047	947	(27.9)	974	(28.7)	0.018
HTN	2,197	(52.8)	3,444	(51.7)	0.034	1,758	(51.9)	1,737	(51.2)	0.012
Dyslipidemia	2,241	(53.8)	3,268	(49.0)	0.096	1,810	(53.4)	1,776	(52.4)	0.020
CVD	164	(3.9)	373	(5.6)	0.078	139	(4.1)	140	(4.1)	0.001
AF	116	(2.8)	200	(3.0)	0.013	92	(2.7)	91	(2.7)	0.002
COPD/Asthma	156	(3.7)	297	(4.5)	0.036	132	(3.9)	128	(3.8)	0.006
Dementia/Parkinsonism	37	(0.9)	86	(1.3)	0.039	30	(0.9)	34	(1.0)	0.012
OA/RA/Rheumatism	370	(8.9)	674	(10.1)	0.042	309	(9.1)	316	(9.3)	0.007
**CHA_2DS_2-VASc score**										
≥2	3,037	(73.0)	4,952	(74.3)	0.030	2,436	(71.9)	2,468	(72.8)	0.021
0–1	1,126	(27.0)	1,715	(25.7)	0.030	954	(28.1)	922	(27.2)	0.021
**Medication use**										
ACEI/ARB	3,390	(81.4)	5,402	(81.0)	0.010	2,760	(81.4)	2,755	(81.3)	0.004
Beta-blockers	3,270	(78.5)	4,827	(72.4)	0.143	2,609	(77.0)	2,605	(76.8)	0.003
Nitrates	3,826	(91.9)	6,104	(91.6)	0.013	3,112	(91.8)	3,078	(90.8)	0.036
Aspirin	4,163	(100.0)	6,667	(100.0)	<0.001	3,390	(100.0)	3,390	(100.0)	<0.001
$P2Y_{12}$ inhibitors	4,163	(100.0)	6,667	(100.0)	<0.001	3,390	(100.0)	3,390	(100.0)	<0.001
Statins	3,664	(88.0)	5,289	(79.3)	0.237	2,951	(87.1)	2,969	(87.6)	0.016
PPIs	404	(9.7)	706	(10.6)	0.029	315	(9.3)	331	(9.8)	0.016
Steroids	345	(8.3)	736	(11.0)	0.093	296	(8.7)	300	(8.8)	0.004
NSAIDs	1,124	(27.0)	2,314	(34.7)	0.167	987	(29.1)	911	(26.9)	0.050
**ORBIT score**										
≥3	62	(1.5)	178	(2.7)	0.083	49	(1.4)	50	(1.5)	0.002
0–2	4,101	(98.5)	6,489	(97.3)	0.083	3,341	(98.6)	3,340	(98.5)	0.002
**ARC criteria of bleeding risk**										
**Major criteria**										
Malignancy	112	(2.7)	129	(1.9)	0.050	66	(1.9)	70	(2.1)	0.008
Long-term use of anticoagulants	42	(1.0)	100	(1.5)	0.044	34	(1.0)	35	(1.0)	0.003
End-stage CKD	10	(0.2)	11	(0.2)	0.017	8	(0.2)	4	(0.1)	0.028
ICH	18	(0.4)	43	(0.6)	0.029	17	(0.5)	19	(0.6)	0.008
**Minor criteria**										
Age ≥75	415	(10.0)	866	(13.0)	0.095	317	(9.4)	312	(9.2)	0.005
Ischemic stroke	82	(2.0)	201	(3.0)	0.067	67	(2.0)	68	(2.0)	0.002

Bleeding events requiring hospitalization or transfusion	33	(0.8)	107	(1.6)	0.075	28	(0.8)	41	(1.2)	0.038
**DAPT at discharge of index MI**										
Aspirin	4,163	(100.0)	6,667	(100.0)	<0.001	3,390	(100.0)	3,390	(100.0)	<0.001
$P2Y_{12}$ inhibitors	4,163	(100.0)	6,667	(100.0)	<0.001	3,390	(100.0)	3,390	(100.0)	<0.001

Abbreviations: ACEI = angiotensin-converting enzyme inhibitor; ARB = angiotensin II receptor blocker; ARC = Academic Research Consortium; AF = atrial fibrillation; BMS = bare-metal stent; CHA_2DS_2-VASc score = congestive heart failure, hypertension, age ≥75 years, diabetes, stroke/transient ischemic attack, vascular disease, age 65 to 74 years, female sex; CKD = chronic kidney disease; COPD = chronic obstructive pulmonary disease; CVD = cerebrovascular disease; DES = drug-eluting stent; DM = diabetes mellitus; HTN = hypertension; IABP = intra-aortic balloon pump; ICH = intracranial hemorrhage; NG-DES = newer-generation drug-eluting stent; NSAIDs = nonsteroidal anti-inflammatory drugs; OA = osteoarthritis; OACs = oral anticoagulants; ORBIT score = age ≥ 75 years, bleeding history, chronic kidney disease, treatment with antiplatelet; PCI = percutaneous coronary intervention; PPIs = proton pump inhibitors; PSM = propensity score matching; RA = rheumatoid arthritis; SD = standard deviation; SMD = standardized mean difference; STEMI = ST-elevation myocardial infarction;

*SMD = difference in means or proportions divided by standard error and imbalance defined as an absolute value greater than 0.1.

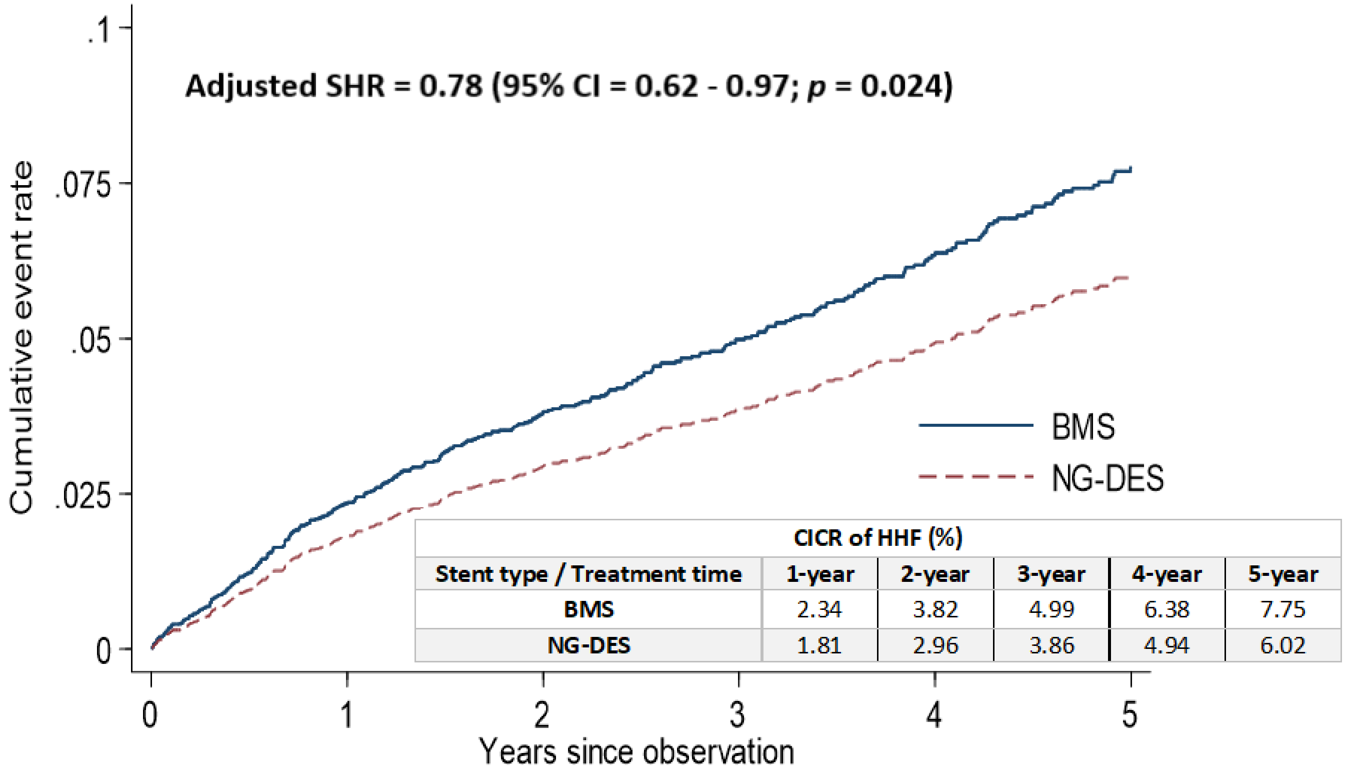

CICR of HHF (%)					
Stent type / Treatment time	1-year	2-year	3-year	4-year	5-year
BMS	2.34	3.82	4.99	6.38	7.75
NG-DES	1.81	2.96	3.86	4.94	6.02

Figure S2. Cumulative incidence of HHF for competing risk among patients with STEMI who received DAPT for an undefined duration of longer than 6 months following stent implantation.

Abbreviations: BMS = bare-metal stent; CI = confidence interval; CICR = cumulative incidence for competing risk; DAPT = dual antiplatelet therapy; HHF = hospitalization for heart failure; NG-DES = new-generation drug-eluting stent; STEMI = ST-elevation myocardial infarction; SHR = subdistribution hazard ratio.

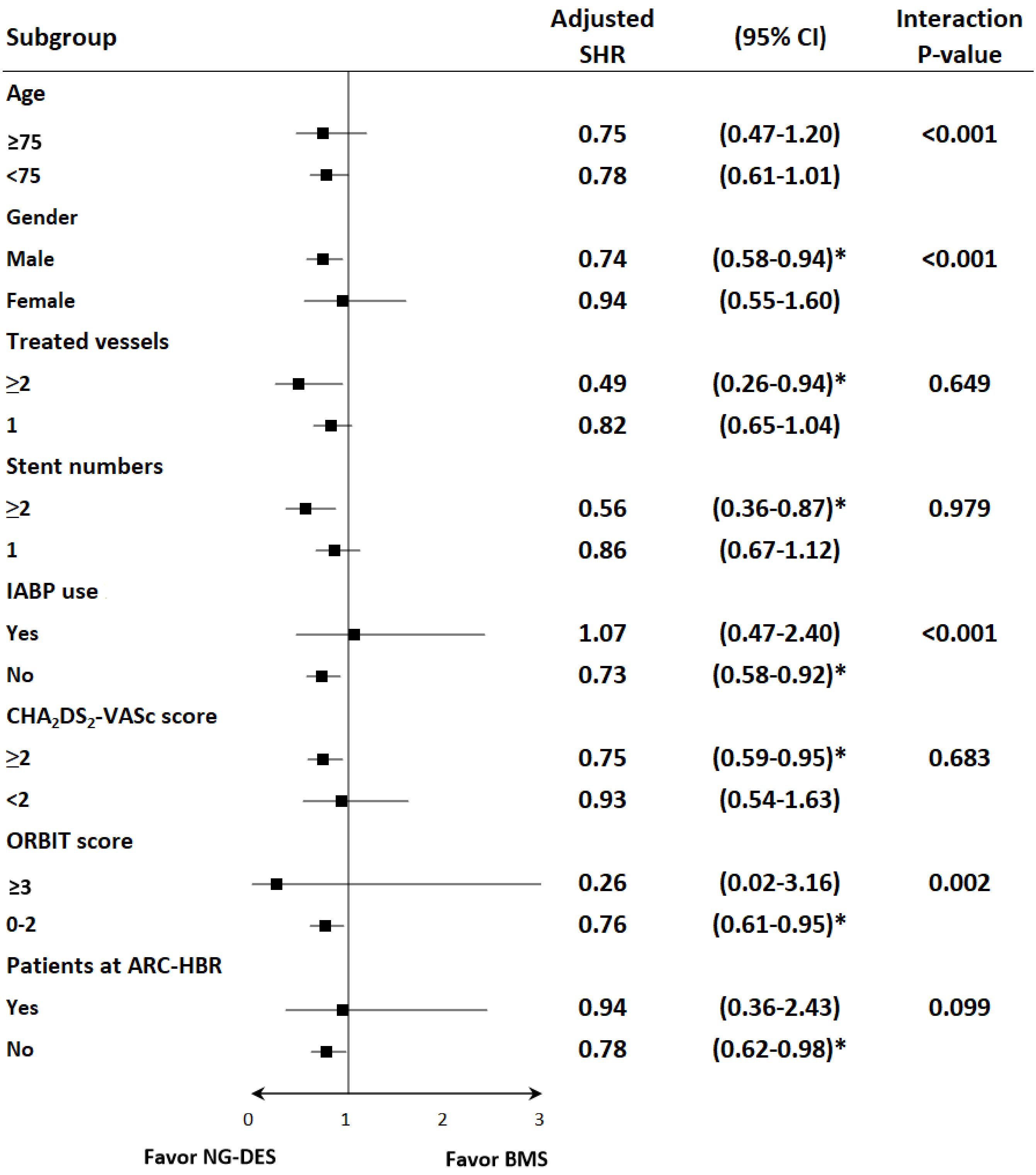

Figure S3. Subgroup analysis of the risk of HHF among patients with STEMI who received DAPT for an undefined duration of longer than 6 months following stent implantation. Abbreviations: BMS = bare-metal stent; CHA_2DS_2-VASc score = congestive heart failure, hypertension, age ≥ 75 years, diabetes, stroke/transient ischemic attack, vascular disease, age 65 to 74 years, female sex; CI = confidence interval; HHF = hospitalization for heart failure; IABP = intra-aortic balloon pump; NG-DES = new-generation drug-eluting stent; STEMI = ST-elevation myocardial infarction; ORBIT score = age ≥ 75 years, bleeding history, chronic kidney disease, treatment with antiplatelet; PCI = percutaneous coronary intervention; SHR = subdistribution hazard ratio.

* p <0.05

**Table S4. The rates of CICR and the risk of repeat PCI in patients with STEMI receiving NG-DESs compared with those patients with STEMI receiving BMSs.**			
**Stent types**	**5-year CICR**	**Adjusted SHR (95% CI)**	***p***
**BMS**	39.7%	Reference	-
**NG-DES**	34.9%	0.85 (0.80 – 0.90)	<0.001

Abbreviation: BMS = bare-metal stent; CI = confidence interval; CICR = cumulative incidence competing risk; NG-DES = new-generation drug-eluting stent; PCI = percutaneous coronary intervention; SHR = subdistribution hazard ratio; STEMI = ST-elevation myocardial infarction

**Table S5. The rates of CICR and the risk of recurrent MI in patients with STEMI receiving NG-DESs compared with those patients with STEMI receiving BMSs.**			
**Stent types**	**5-year CICR**	**Adjusted SHR (95% CI)**	***p***
**BMS**	12.2%	Reference	-
**NG-DES**	10.0%	0.80 (0.72 – 0.90)	<0.001

Abbreviation: BMS = bare-metal stent; CI = confidence interval; CICR = cumulative incidence competing risk; MI = myocardial infarction; NG-DES = newer-generation drug-eluting stent; SHR = subdistribution hazard ratio; STEMI = ST-elevation myocardial infarction.

Table S6. Baseline characteristics of patients with STEMI who were at ARC-HBR and non-ARC-HBR after PSM.

	**ARC-HBR*** **N= 781**	**Non-ARC-HBR N = 12,881**	***p***
**Age, mean±SD**	68.6 ± 12.6	58.7 ± 12.1	<0.001
**Male, n (%)**	622 (79.6)	11,356 (88.2)	<0.001
**Use of NG-DES, n (%)**	387 (49.6)	6,444 (50.0)	0.79
**Major ARC criteria of bleeding risk**			
- **Malignancy**	353 (45.2)	0	-
- **Long-term use of anticoagulants**	195 (25.0)	0	-
- **End-stage CKD**	34 (4.4)	0	-
- **ICH**	72 (9.2)	0	-
**Minor ARC criteria of bleeding risk**			
- **Age ≥75**	303 (38.8)	1,473 (11.4)	<0.001
- **Ischemic stroke**	154 (19.7)	245 (1.9)	<0.001
- **Bleeding events requiring hospitalization or transfusion**	69 (8.8)	122 (0.9)	<0.001
**DAPT adherence, n (%)**			
- **At discharge**	768 (98.3)	12,759 (99.1)	0.071
- **1 month after discharge**	564 (72.2)	11,487 (89.2)	<0.001
- **3 months after discharge**	463 (59.3)	9,155 (71.1)	<0.001
- **6 months after discharge**	347 (44.4)	6,760 (52.5)	<0.001
- **12 months after discharge**	159 (20.4)	2,973 (23.1)	0.075

Abbreviations: ARC = Academic Research Consortium; CKD = chronic kidney disease; DAPT = dual antiplatelet therapy; DES = drug-eluting stent; HBR = high bleeding risk; ICH = intracranial hemorrhage; NG-DES = new-generation drug-eluting stent; PSM = propensity score matching; SD = standard deviation; STEMI = ST-elevation myocardial infarction.

*Patients were defined to be at ARC-HBR if at least 1 clinical major or 2 minor ARC criteria of bleeding risk are met.
